# Impact of aloe vera on post-surgical wound healing in chronic periodontitis patients undergoing periodontal flap surgery: A randomized controlled trial

**DOI:** 10.6026/973206300213309

**Published:** 2025-09-30

**Authors:** Manvi Chandra Agrawal, Indrani Bharadwaj, Prerna Agarwal, Ashutosh Agarwal, Geetika Kumar, Somya Tiwari

**Affiliations:** 1 Department of Periodontology and Implantology, Institute of dental sciences Bareilly, Uttar Pradesh, India

**Keywords:** Aloe vera, flap surgery, wound healing, periodontitis

## Abstract

Periodontal flap surgery is widely performed for pocket reduction, but healing may be delayed by inflammation. Among the recent emergence of herbal and ayurvedic plants, Aloe vera is one such succulent plant which has been used for its anti-inflammatory, anti-microbial properties and it also may enhance surgical outcomes. Therefore, it is of interest to assess the effect of topical aloe vera gel on postoperative healing, probing pocket depth and clinical attachment loss following periodontal flap surgery. Hence, thirty patients with Stage II/III periodontitis were randomly assigned into test (aloe vera gel) and control (Co-E pack) groups. Flap surgeries were conducted on the patients. Postoperative healing was assessed using the Early Healing Index (EHI) at one week and the Healing Index (HI) at one, two and three weeks. PPD and CAL were recorded pre- and postoperatively. The aloe vera group showed significantly higher EHI scores at one week and higher HI scores at week two and three. Both groups improved over time, but PPD reduction and CAL gain were greater with aloe vera. Superior healing was seen in the aloe vera group regardless of disease severity. Thus, topical aloe vera gel significantly improved postoperative healing, PPD and CAL following flap surgery, suggesting its potential as a safe, effective adjunct in periodontal therapy.

## Background:

Chronic periodontitis is an infectious disease which results in inflammation of the supporting and surrounding structures of the teeth and leads to bone loss [1]. It significantly affects quality of life as well as general well-being among patients [2]. The various treatments for periodontal diseases varies according to the severity where for minor cases scaling and root planning is done, however in advanced cases surgical interventions such as flap surgery which requires careful repositioning of the gingival connective tissue on the tooth surface that has been prepared is required. Flap surgeries although effective in controlling disease progression and improving long term treatment outcomes, often leads to considerable soft tissue manipulation which results in post-surgery discomfort, delayed wound healing with elevated risk of infection [3]. Therefore, accelerating wound healing in periodontal surgeries remains a key goal of clinicians. While conventional therapies are effective, there is growing interest in adjunctive natural agents that can enhance healing, reduce inflammation and minimize postoperative discomfort. Among such agents, Aloe vera has attracted significant attention due to its multifaceted therapeutic properties [4]. Aloe vera (Aloe barbadensis Miller) belonging to the Lilaceae family has been documented to have excellent pharmacological properties which includes hypoglycemic effects, anti-inflammatory, antibacterial, antioxidant, antiviral and antifungal effects. Studies have found aloe vera to be beneficial in dentistry as its antibacterial properties may help reduce the microbial load in the surgical site, potentially preventing secondary infections that can impair healing outcomes [5].

The clear gel obtained from the leaves of the plant are rich in biologically active compounds including acemannan - a polysaccharide, vitamin A, C, E and B12, enzymes, amino acids and minerals. These components have been shown to enhance fibroblast activity, promote collagen synthesis, which are required for effective wound healing. In the context of oral health, several studies have reported the beneficial role of Aloe vera in managing recurrent aphthous ulcers, oral lichen planus, gingivitis and radiation-induced mucositis. Its topical application in minor wounds and burns has also shown significant improvement in healing rates [6]. Given the challenges associated with wound healing in periodontal flap surgeries, there is a growing interest on adjunctive therapies that can enhance tissue regeneration and patient comfort. In this context, the use of natural agents such as aloe vera offers promising potential. With its well-documented anti-inflammatory, anti-microbial and wound healing properties, aloe vera represents a biologically active, non-invasive and biocompatible option that aligns with current trends in integrative and holistic periodontal care [7, 8]. Although, the benefits are many there is a scarceness of robust clinical evidence evaluating its effectiveness in enhancing wound healing following periodontal surgical procedures, particularly among patients with chronic periodontitis-where healing may already be compromised due to persistent inflammation and altered host immune responses. Therefore, it is of interest to assess the effect of topical aloe vera application on wound healing following periodontal flap surgery in patients with chronic periodontitis.

## Materials and Methodology:

## Study design:

The current study was a split mouth, randomized controlled trial which was carried out at the Department of Periodontology and Implantology, Institute of Dental Sciences, Bareilly, Uttar Pradesh, India. Ethical permission was obtained prior to starting the study (IEC/304/2024/05) and registration at Clinical Trials Registry - India was also done (Ref/2025/05/105902). The study confirms to the guidelines set by the Consolidated Standards of Reporting Trials (CONSORT) [9].

## Eligibility criteria:

The current study population comprised individuals belonging to the age between 25 to 55 years who were diagnosed with chronic generalised periodontitis. Eligible participants were those presenting with periodontal pocket depth measuring 5 mm or more and demonstrating good oral hygiene maintenance. Only systemically healthy individuals who were fit to undergo periodontal surgery were considered. Furthermore, the study focused specifically on patients classified as having Stage II or Stage III periodontitis [10] ensuring a consistent level of disease severity across participants. Individuals were excluded from the study if they had any current or prior systemic disorders which might affect the outcome of periodontal treatment. This included patients with compromised immune systems or those on medications that could interfere with periodontal treatment outcomes. Pregnant women and individuals who smoked were also excluded, given the known influence of these factors on periodontal healing. Additionally, patients with a known allergy to aloe vera were not included in the study to prevent potential adverse reactions.

## Sample size:

An a priori sensitivity analysis was performed in G*Power (v3.1) which indicated that, with α = 0.05, 80% power and equal allocation (n =30), the minimum detectable effect was Cohen's d = 0.72.

## Preparation of aloe vera:

From the plant's leaf pulp from live plants, aloe vera extract was extracted as a viscous gel with the help of sterilized scalpel, maintaining the sterility of the extract. The surface of the aloe vera leaves was cleaned with 70% ethanol to eliminate external microbes. After extraction, the gel was promptly placed into a pre-sterilized, airtight container to avoid environmental exposure. The extract was further stored at 4°C to preserve bioactive compounds like vitamin C and E, polysaccharides like acemannan and antioxidants and also to limit microbial growth. Patients were instructed to use the Aloe vera extract within 24-48 hours to maintain its sterility and therapeutic effectiveness. To prevent its psychological effects, patients were blinded to the effects of aloe vera gel.

## Study procedure:

Following Phase I therapy where scaling, root planing and oral hygiene instructions were done, patients were re-evaluated after four weeks. Thirty patients who met the inclusion criteria were recruited and randomly allocated into test and control groups using a lottery method. Patients diagnosed with Stage II and Stage III periodontitis were included in the study. Both groups underwent periodontal flap surgery for pocket reduction. Flap approximation was achieved using 3-0 non-absorbable black silk sutures. In the test group, patients were instructed to apply aloe vera gel topically to the surgical site, where sutures were placed, three times daily. The gel was applied gently with the fingertip for 10 minutes without massaging. Patients were advised to avoid rinsing or eating for at least one hour following application. All patients were prescribed postoperative medications, including nonsteroidal anti-inflammatory drugs (ibuprofen 400 mg + paracetamol 325 mg) twice daily for three days and antibiotics (amoxicillin 500 mg every eight hours for five days). Patients were advised to refrain from tooth brushing for the first two weeks. Instead, the surgical site was to be cleaned using wet gauze until day 14. One week after surgery, sutures were removed from the surgical site and the area was carefully cleaned with betadine and normal saline. Thereafter, patients were instructed to maintain oral hygiene using an ultra-soft toothbrush with the roll-on technique. Postoperative wound healing was assessed by the same surgeon using the Early Healing Index [11] at one week and the Healing Index [12] at one, two and three weeks following surgery.

## Statistical analysis:

Data thus collected was entered into Microsoft excel sheets and analysis was carried out using SPSS version 23.0. To assess the normality of the data, Shapiro Wilk test was conducted. Unpaired t test was done to draw comparison between the groups. A p value of <0.05 was considered to be statistically significant.

## Results:

A total of 30 participants were present for final analysis in the current study. Thirty sites were from the test group where aloe vera gel was applied and thirty sites where Coe - pack was applied. The comparison of postoperative healing outcomes in Table 1 (see PDF) between the two groups demonstrated that the aloe vera group exhibited significantly better early healing scores (EHI) at one week compared to the Co-E pack group (p < 0.001). With respect to the Healing Index, no significant difference was observed between the groups at one week postoperatively; however, by the second week, the aloe vera group showed significantly higher healing scores than the Co-E pack group (p = 0.010). This trend persisted at the third week, where the aloe vera group again demonstrated superior healing outcomes (p = 0.001). Pairwise comparison of periodontal parameters revealed significant improvements in both groups (Table 2 -see PDF). In the aloe vera group, a marked reduction in probing pocket depth (PPD) was observed from baseline (4.93 ± 0.96) to the postoperative period (0.13 ± 0.35), along with a significant gain in clinical attachment level (CAL) from 6.00 ± 1.00 at baseline to 0.67 ± 0.72 postoperatively (p < 0.001 for both). Similarly, in the Co-E pack group, PPD decreased significantly from 1.60 ± 0.51 at baseline to 1.00 ± 0.00 postoperatively, while CAL improved from 4.33 ± 1.40 to 2.53 ± 0.52 (p < 0.001 for both). These findings indicate that both treatment modalities contributed to significant reductions in PPD and gains in CAL, with the aloe vera group showing a more pronounced improvement. At the third postoperative week, healing outcomes varied according to the severity of periodontitis and treatment group as shown in Table 3 (see PDF). In the aloe vera group, patients with moderate periodontitis showed favorable healing, with 50% achieving a healing score of 4.00 and the remaining 50% attaining the highest score of 5.00. Among those with severe periodontitis, 42.9% achieved a healing score of 4.00, while 57.1% reached a score of 5.00, indicating consistently better wound healing. In contrast, the Co-E pack group demonstrated poorer outcomes. In patients with moderate periodontitis, the majority (75%) had a healing score of 3.00 and only 25% reached a score of 4.00, with none achieving the maximum score. Similarly, among those with severe periodontitis, 87.5% remained at a healing score of 3.00, while only 12.5% reached a score of 4.00 and none attained a score of 5.00. These findings suggest that aloe vera was associated with superior healing outcomes compared to the Co-E pack, irrespective of disease severity.

## Discussion:

The present randomized clinical trial evaluated the effect of topical aloe vera application on postoperative healing following periodontal flap surgery. Our findings revealed that patients in the aloe vera group demonstrated significantly superior early healing scores (EHI) after one week and higher healing scores (HI) at the second and third postoperative weeks compared to those in the Co-E pack group. Additionally, marked decrease in probing pocket depth (PPD) improvements in clinical attachment level were noted in the aloe vera group, indicating enhanced periodontal tissue recovery. Notably, even in patients with severe periodontitis, aloe vera promoted higher healing scores at the third week compared to the control group. These improved outcomes can be explained by the multiple pharmacological benefits of aloe vera. It contains biologically active compounds such as acemannan, glucomannans, vitamins and minerals, which have been shown to stimulate fibroblast proliferation, collagen synthesis and angiogenesis-processes crucial for periodontal wound healing. Its anti-inflammatory effects, mediated through suppression of cyclooxygenase pathways and prostaglandin E_2_ production, likely reduced tissue inflammation, contributing to faster resolution of edema and discomfort. Furthermore, aloe vera's antimicrobial activity against common periodontal pathogens may have provided a more favourable environment for tissue repair, thereby explaining the observed improvements in both PPD and CAL [13, 14]. Our results are consistent with previous research on aloe vera in periodontal therapy.

A meta-analysis of randomized controlled trials reported significant improvements in PPD and gingival index when aloe vera gel was used as an adjunct to scaling and root planing (SRP) (mean difference in PPD: -0.45 mm; p = 0.009 [15]. Similarly, another study showed adjunctive use of aloe vera gel as local drug delivery shows greater gains in clinical attachment in severe periodontal cases [16]. A more recent trial by Katariya *et al.* [17] confirmed that SRP combined with aloe vera hydrogel was superior to SRP alone in improving periodontal parameters. Our study extends these findings into the surgical domain, demonstrating that aloe vera is equally effective in enhancing postoperative healing following flap procedures. Furthermore, the association between disease severity and healing response observed in our study provides additional clinical relevance. While Co-E pack patients, particularly those with severe periodontitis, exhibited limited healing by the third week, the aloe vera group consistently achieved higher healing scores regardless of baseline severity. This suggests that aloe vera's regenerative and anti-inflammatory properties may help overcome the impaired healing typically associated with advanced periodontal destruction. Nevertheless, certain limitations must be acknowledged. The sample size was modest (n = 30), which, while sufficient to detect large effects, may not fully capture interindividual variability. The follow-up period was restricted to three weeks and long-term stability of PPD and CAL gains remains to be determined. Histological evidence of healing was also not assessed. Future multicenter RCTs with larger sample sizes, longer follow-up and adjunctive histological or microbiological analyses are warranted to validate and expand upon these findings.

## Conclusion:

Within the limitations of this study, the application of fresh Aloe-vera gel was effective in improving healing scores in the 1st postoperative week. Thus, Aloe-vera is an effective and low cost therapeutic agent for better periodontal wound healing.
